# Royal Infirmary of Edinburgh

**Published:** 1829-01-01

**Authors:** 


### ROYAL INFIRMARY OF EDINBURGH.

### I. FRACTURES AND DISLOCATIONS.

To the surgeon *par excellence*, and the general practitioner, the diagnosis and management of fractures and dislocations must ever be a subject of paramount importance. Commonly, nay constantly, occurring, often in the persons of the powerful and rich, the medical attendant is generally required to act upon the moment, and not seldom to stake his reputation on the issue. Cases of fracture mistreated, or thought to be mistreated, form far the greatest share of the legal prosecutions instituted by patients against medical practitioners; and how often, on the other hand, has a young man in the country established his fame, and filled his pockets too, by setting a bone with success! All who are engaged in, and most who are acquainted with the practice of our hospitals, must have witnessed again and again, the most lamentable specimens of ignorance and blunders, sent, as mementos of the *skill* of the *doctor*, and received, alas! too late for any useful purpose, except

> To point a moral, or adorn a tale!

Under the impression that the subject is one of great practical utility, we purpose returning to it every now and then, as opportunities present themselves. In another part of our Hospital Practice department, will be found the results of Dr. Anderson's experience at the Glasgow Infirmary; and now we shall present to our readers some cases and remarks from the practice and pen of Dr. Ballingall, in the similar institution of Edinburgh.

CASE 1. *Double Fracture of the Tibia—imperfect Union.* Donald Clark, ætatis 72, on the 26th of March, had his leg jammed between a coal cart and a wall, and, soon after, was admitted into the Infirmary under the care of Dr. Ballingall. The tibia was fractured an inch and a half below its tubercle, and again two inches lower down, the intermediate bone being moveable and loose; the fibula was broken nearly opposite the lower fracture of the tibia. The limb was placed "on M'Intyre's splint, with a pasteboard splint on either side, firmly confined with a roller." On the 10th of May, the upper fracture had united, but the lower one crepitated still. Beef, porter, &c. were ordered; but, four months elapsing, and mobility of the fracture remaining still, the patient was dismissed. The limb, at this time, was secured in pasteboard splints, and strong injunctions given to move, as much as possible, with the assistance of crutches, for the purpose of exciting action in the seat of the fracture. Some little time after dismissal, the patient was seen by the house surgeon, who reported the union as slowly advancing, though still incomplete.

Dr. Ballingall believes, that non-union depended on a general defect of constitution, which appeared much impaired by hard labour, and evinced the accession of a premature, rather than extreme old age. This may be true; but why should a bad constitution act only at the distance of four inches below the tubercle of the tibia, and be perfectly innocuous at two? Depend upon it, a local cause was, also, superadded to the general one, that local cause being probably the fracture of the fibula. When the tibia only is broken, the fibula acts as an excellent splint, a splint which is superior, even to "Mr. M'Intyre's." The *upper* fracture, in this case, was solely of the tibia, and union took place *malgré* the defects of the system at large. The lower fracture implicated tibia *and* fibula, wherefore the process was infinitely slower. We do not overlook, nor indeed underrate the debility of the constitution, the main cause, perhaps, after all; but still we believe that the circumstance here pointed out, must also be weighed in the scale.

CASE 2. *Ununited Fracture of the Humerus—Ends of Bone removed.*—Thomas Christie, aged 41, was admitted under the care of Mr. Liston, on the 9th of June, with transverse fracture of the humerus, four inches above the elbow. The ends of the bone overlapped each other, the lower one passing in front; no union had occurred, but extension was inoperative in bringing the extremities into apposition. He stated that six weeks before, whilst at work in a pit, a piece of wood fell and struck him on the arm. A good deal of swelling and inflammation succeeded, for which he was bled, and, at different periods, had leeches applied to the number of eighty. Three weeks after the accident, splints were applied, which he says were repeatedly obliged to be removed, on account of the swelling and inflammation. The limb, since the accident, had been kept in a bent position. The first thing which was done by Mr. L. was bandaging the fore-arm with flannel, the use of a paste-board splint on each side of the arm, and a wooden one on the outside firmly applied over all. A beef-steak and a bottle of porter were given daily, and the splints continued on the arm till the 30th, at which time they were removed, no union or consolidation whatever, having occurred. A portion of each broken extremity of the bone was obliquely cut away by Mr. Liston; a splint, which supported the fore-arm, applied on the inside of the arm, and a short splint again on the outside. Much swelling and tension succeeded the operation, which rendered it necessary to remove the outer splint; but the constitutional disturbance appears to have been slight. A collection of matter, which formed over the region of the biceps muscle, kept open for some time the centre of the wound; but this had nearly healed, and all was doing well, when the patient unfortunately fell out of bed. The process of union, though disturbed, will not, Dr. Ballingall trusts, be ultimately stopped.

"In speaking of this case, I noticed a number of others in which this operation had been practised with various degrees of success; I fear, however, that I omitted to mention, or to give you a reference to two interesting cases treated some years ago in this house, one by the late Dr. Wardrop, and the other by my colleague Dr. Inglis. Of these two cases you will find the particulars detailed in the 1st volume of the Edinburgh Medical and Surgical Journal; and what renders them the more remarkable is, that in both instances union was procured, although the fracture existed in the one case in the fore-arm, and in the other in the leg; situations in which Boyer and other eminent surgeons had altogether discountenanced the performance of this operation."

Some little time ago a case was to be seen at St. George's Hospital, which excited much attention, and put the surgeons and instrument makers to their very wits' ends. It was that of a woman with ununited fracture of the humerus. Compression by the tourniquet, a seton between the ends of bone, and various mechanical apparatuses and contrivances were marshalled in the field, but all to no effect. The patient was of a debilitated and irritable habit. Before quitting the subject of ununited fractures, we may mention a circumstance deserving of attention. When the process of union appears to be slow, a blister placed opposite the seat of the fracture occasionally proves of much service.

CASE 3. *Complete Dislocation of the Elbow-Joint, mistaken for Fracture.*—Dorcas Spiers, ætatis 6, was admitted on the 4th of June in the following condition.

" 'On either side of the upper arm there is a pasteboard splint, tightly confined by a roller, the lower part of the limb very tense, and somewhat discoloured, the fore-arm has not, according to the usual method of treatment, been placed at a right angle with the upper arm, but forms an obtuse angle with it, and cannot be either bent or extended farther; upon the removal of the splints, the head of the radius was found lying behind the outer condyle of the humerus; the entire upper surface of the olecranon was felt through the soft parts, and there was a distinct depression above this process of the ulna; the humerus was indistinctly felt lying on the anterior surfaces of the bones of the fore-arm; crepitus was quite evident, but appeared to arise entirely from the friction of the displaced bones upon each other, and no crepitus could be detected when the two extremities of the humerus alone were moved in opposite directions. The dislocation was reduced by bending and extending the fore-arm over the knee. The fore-arm was afterwards bent to a right angle with the upper arm, and supported by a sling, a cold lotion being applied to the inflamed parts."

On the 12th, passive motion was employed, and the lad was, on the 16th, dismissed quite cured. The nature of the case had been evidently mistaken, although the characteristic marks of the dislocation would appear to have been tolerably clear. It would be well for the profession, though not for that part of it whose bread is abuse, and who could no more subsist without slander, than the vampyre without blood, if the following sentiment were generally felt and universally acted on. "Although the error in this case appears to have been a gross one, I would not willingly be thought the last to find an excuse for it. It has been well observed, that it becomes us on all occasions to weigh errors in the balance of good will, and rather to point them out for amendment than for censure."

Case 4. *Dislocation of the Radius backwards*. William Henderson, aged 12, was admitted on the 30th of March, having received the following injury to the elbow-joint, from a fall, four days previously, on the palm of the hand. The soft parts about the joint were much swelled—the skin inflamed, vesicated in some parts, abraded in others—the fore-arm immoveably pronated, and forming an obtuse angle with the upper arm—flexion and extension exceedingly confined. The external condyle of the humerus was very indistinct, and the head of the radius was felt lying behind it, whilst the tip of the finger could be placed in its articular cavity.

Fearful that the joint had sustained other injury, besides the displacement of the head of the radius, leeches were applied, and reduction postponed for a couple of days. Satisfied, at this time, that only the radius was dislocated backwards, it was readily replaced by extending the fore-arm, bending it on the upper arm, and pronating the radius. The fore-arm, after this, was for some time supported in a sling, passive motion employed, and the boy, in a short time, dismissed the infirmary.

In the following notes of a dissection, the head of the radius is seen to continue dislocated backwards, although the olecranon would seem to have regained its natural position. All the ligaments were strong, and little altered in direction. The outer condyle of the humerus was rough and irregular at the spot where the radius naturally plays upon it, whilst its lower and back part presented an articulating, cartilage-tipped surface, receiving the upper extremity of the radius, which was rounded, enlarged, and likewise tipped with cartilage. The surface of the humerus naturally forming the articulation with the ulna, was rough and degenerated before, but hollowed behind, where the coronoid process had pressed on it. The "olecranoid cavity" was elongated, the olecranon shortened, and appearing to have fallen into its natural position, partly by absorption of its own surface, and partly by absorption of the coronoid process.

We have given these cases at some little length, because Sir Astley Cooper has never seen the accident, dislocation of the head of the humerus backwards, but once, and that in a body brought to the dissecting room.

"The only instance in which I have seen this dislocation, was in a subject brought to St. Thomas's dissecting-room, in the year 1821; the displacement had existed some time. The head of the radius was thrown behind, and to the outside of the external condyle of the humerus, where it formed a projection that could readily be seen as well as felt when the arm was extended. The oblique and coronary ligaments were torn through, and the capsular ligament was partially lacerated. Of the cause of this accident, I am ignorant, as I have never seen the accident in the living body."—*Sir A. Cooper's Lect. Vol.* iii.

We believe that an example has lately been shewn to Sir Astley, by Mr. Fernandez of Lamb's Conduit-street; and Dr. Ballingall has seen two well marked cases of the accident in the course of the last six months. One has already been detailed; the other was that of a young girl from Ayrshire, in whom it had continued unreduced, for a period of thirteen weeks. With regard to the reduction, it is easily effected by bending, and at the same time pronating the fore-arm, which should afterwards be kept at right angles with the arm, till the ligaments, &c. have had time to unite.

### II. Chronic Affections of the Joints.

The frequency of chronic affections of the joints, renders their accurate diagnosis and treatment a matter of importance in every point of view. To Mr. Brodie, the profession owe a debt of obligation, for the excellent work he has written on the subject, a work which deserves to be carefully studied by all who are engaged in either medical or surgical practice.

Some cases of *morbus coxarius*, a vague and highly objectionable term, are detailed by Dr. Ballingall.

Case 1. *Incipient Disease of the Hip —great relief.*—A girl, aged 12, came into the hospital, complaining of pain on the inside of the left thigh and knee, increased upon exercise, and attended with loss of the motions of the limb, especially flexion and extension. The buttock was flattened, the thigh inclined forwards, and the limb slightly lengthened, apparently from the pelvis inclining to that side. The disease was only of three weeks' duration, and began without evident cause. The treatment consisted in leeching, fomentations, and confinement to the horizontal position. At the end of six weeks, the disease having yielded, the patient was dismissed.

Case 2. *Disease of the Right Hip-Joint—Elongation of the Limb.* In this case, the disease had advanced further than in the former, and presented the characters of ulceration of the cartilages. The patient was a girl of 17, and stated that, eight months before her admission, she began to be affected with stiffness and pain in the joint, which gradually increased for the first seven months, but with greater rapidity during the last. She suffered at times throbbing pain in the hip, and pressure on the trochanter, or motion of the limb, was also productive of pain. No swelling or redness were present—the limb appeared lengthened for an inch and a half—the toes were everted. The spine was slightly curved towards the left in the lower dorsal vertebræ, the anterior superior spinous process of the ilium higher on that side than the opposite. The limb was easiest when extended; the general health good.

Leeches were applied in the first instance, and moxas employed contiguous to the joint. On the 13th, a blister was placed on the groin and inside of the thigh, and the patient at present has been partially relieved.

Caustic issues, we think, answer better than moxas in recent affections of the joints, though very much depends on the proper mode of using them. If the beans are kept in for a week or ten days at a time, even though the depth of the issue should be maintained, it is really of very little service. The sore is in an indolent, we might almost say callous condition; the discharge, for the most part, is sparse; the sensibility of the surface but trifling; and the object of an active counter-irritation defeated. The best plan, and that recommended by Mr. Brodie in his Clinical Lectures, is to discard the employment of the beans altogether, rubbing the issue every two or three days with the potash, and keeping up a moderate pressure with lint in the intervals. If the surface of the issue looks sloughy or inflamed, a poultice should always be applied, as such a condition of the superficial sore is sometimes productive of great irritation.

"In the treatment of the morbus coxarius, our object is to subdue the inflammatory action within the joint, and this we attempt in the early and acute state of the disease by the repeated application of leeches; and subsequently by the use of what are termed counter-irritants, blisters, moxas, caustic issues, and setons. This last class of remedies may perhaps be employed here with less equivocal effects, and with a better prospect of success, than in other joints more superficially covered, where it appears to me doubtful how far we can establish an inflammatory action on the surface without the risk of its extending to the interior. Whatever affords a rational prospect of relief, ought to be employed with particular assiduity and perseverance in diseases of the hip-joint, because the removal of the limb, to which we often resort with success in the diseases of other joints, is here scarcely admissible, or at least its practice in this disease has not been encouraging. The excision of the head of the bone in some aggravated cases, where the articular apparatus has been destroyed, and the joint dislocated, is, in my opinion, a much more promising operation; and I mentioned to you a very interesting case, in which it was performed successfully by Schmalz, and which you will find detailed by Hedenus of Dresden in an excellent thesis, '*De femore in cavitate cotyloidea amputando*.'"

Never having perused Hedenus's thesis, we are unable to form any accurate judgment of the nature of the case where excision was performed. From reason, however, as well as analogy, we believe that this operation would never succeed, in a case so desperate as really to justify, or rather excuse its performance. The consideration of the question would carry us too far beyond our limits to allow us to attempt it here. With one more quotation we conclude.

"With reference to these two last mentioned cases, I offered you some extended observations on the hip-disease, which, from its frequent occurrence and dangerous nature, becomes one of much importance. I showed you that the elongation of the limb which is so marked a feature in the early stages of this affection, although it may be in some slight degree influenced by changes in the joint itself, by swelling of the cartilages, or effusion within the capsule, is in all cases, when it becomes so conspicuous as in Orrock's case, to be explained only by the oblique position which the pelvis assumes. From the tenderness of the affected joint, the patient is induced to throw the weight of his body upon the top of the sound thigh, while the affected limb is generally stretched out, and thrown forwards as a stay to prevent him from falling, and to assist him in progression. The pelvis is thus gradudually and almost imperceptibly elevated on the sound side, while it drops proportionally on the diseased one, and this obliquity often continues to increase until the patient becomes bedridden, when we frequently find the limb shortened, and the position of the pelvis reversed. This appears to me to be in a great measure owing to a continued state of contraction in the psoas magnus muscle of the affected side, which, originating by different slips from the sides and transverse processes of the lumbar vertebræ, passes down to be inserted into the trochanter minor; its contraction, therefore, tends to approximate the transverse processes on the diseased side, and to give a lateral curvature to the lumbar portion of the spine. This is perhaps aided by the patient's lying for the most part on the sound side, resting upon the crest of the ilium, and thus pushing the pelvis towards the opposite side.

"In more advanced stages of the disease, we have a shortening of the limb from circumstances less equivocal, and of which the explanation is abundantly obvious; such are—the destruction of the ligaments, the absorption of the head of the bone, and consequent luxation of the joint—the absorption, in some instances, of the brim of the acetabulum, and in others of the bottom of this receptacle, with the consequent intrusion of the head of the bone into the cavity of the pelvis—of all which I showed you examples.

"Amongst other preparations illustrating the ravages of this disease, I showed you an interesting, and perhaps an unique specimen from Dr. Knox's collection, which gives at once a view of all the consequences above enumerated.

"Here you saw, on the right side, the acetabulum obliterated, and in its site a rough irregular surface, with scarcely any vestige of an articular cavity; the head of the thigh-bone, on this side, divested of its usual globular form, its surface rough, and the limits between it and the cervix femoris very indistinctly marked. On the left side, the os innominatum is smaller than its opposite fellow; in the site of the acetabulum there is a complete breach or perforation in the bone, with some preternatural ossific deposit above it near the anterior and inferior spinous process of the ilium. The head and neck of the femur, on this side, have been completely absorbed as far down as the trochanter major; this process remains, and the intermediate portion of bone between it and the lesser trochanter is rough and quite irregular in its surface; below this point the femur is smooth and shrunk in every dimension; and this wasting or atrophy extends to the other bones of the limb, the tibia and fibula being both shorter as well as smaller than their fellows of the opposite side. Of this singular preparation, I expect we will be furnished with a much more minute and perfect description in a work on the pathology of the bones, by Mr. Benjamin Bell, now in the press. The history of the subject from whom it was taken, a lad apparently about eighteen or twenty years of age, is unfortunately unknown. Dr. Knox has, however, very kindly offered me permission to take a sketch of this preparation, and I give it to you as a valuable and impressive memorandum of the nature and occasional consequences of this destructive disease."

Several other interesting cases and remarks remain to be noticed in a future article.

### XLIV.

### EDINBURGH ROYAL INFIRMARY.

### I. CANCEROUS TUMOURS.

*Case* 1. Martin Smith, ætatis 42, was admitted on the 6th of May with the left side of the nose, the inner halves of both palpebræ, and the soft parts for the depth of half an inch on the inside of the left eye-ball, affected with an ulcer of a carcinomatous character. The periosteum of the ossa nasi and os planum apparently partook of the disease, though the bone was not felt denuded. The vision was perfect—the general health good—he complained of no lancinating pain. The ulcer had commenced in the shape of a small pimple two years before, and progressively got worse.

"This ulcer, Gentlemen, presented, if we except the absence of lancinating pain, all the features of a genuine cancer of the eye, beginning, as that disease usually does, not in the globe of the eye, but in the soft parts surrounding it. In this instance it had involved both the eyelids to a great extent, and had begun to encroach on the sclerotic coat towards the inner or nasal side of the eyeball. Under these circumstances, it became expedient to remove the whole contents of the orbit along with the diseased palpebræ, which I did a few days after his admission; the diseased periosteum covering the bones on the nasal side of the orbit was scraped off with a scalpel, and the cavity filled with charpée."

Healthy granulations quickly appeared, cicatrization succeeded, and before his dismissal on the 19th of June, the sore was reduced to the size of a sixpence. Towards the end of July, Dr. Ballingall received a letter from the patient at Aberdeen, informing him that the eye continued well, the eye-lid shut, and the sore quite healed. In spite of these favourable omens, we do not, for our own parts, feel sanguine respecting the ultimate result.

*Case* 2. The following case is interesting on account of the operation had recourse to for a salivary fistula.

John Adam, ætatis 47, was admitted with a very hard tumour, the size of an egg, situated about an inch in front of the right ear, and nearly on a level with the nose. Its base adhered, but slightly, to the mucous membrane of the mouth, and superiorly it was closely connected with the malar bone and zygomatic arch, above which it rose and lay on the internal angular process of the frontal bone. Over the most prominent part of the tumour, unhealthy ulceration had taken place, and the integuments were purple around. The general health was good—the bowels regular—the pain but slight. He stated that the disease had begun with a small pimple six months previously, and rapidly increased since the 1st of May. He had never been addicted to the use of ardent spirits.

On the 4th of July, the tumour was removed by two elliptical incisions, the masseter muscle being bared, and the os malæ scraped. Two vessels were secured, but secondary hæmorrhage, apparently from the transversalis faciei, occurred in about an hour after the operation, and was stopped by a ligature on the vessel. On examining the tumour it presented "the characters of carcinoma," characters which must ever induce an unfavourably augury in the surgeon's mind.

"In a few days after the removal of this tumour, when the surface left by its excision had assumed a clean, healthy, and granulating appearance, I pointed out to you the orifice of the parotid duct which had necessarily been divided in the operation; this was immediately rendered conspicuous by the flow of saliva on making the patient chew a crust of bread; and it is chiefly with a view to the simple operation which becomes necessary to conduct the secretion of the parotid gland into the mouth when its duct is wounded, that I am at present induced to notice this case. On Saturday last you saw me thrust a small trocar obliquely through the patient's cheek, and after withdrawing the stilette, a small cat-gut bougie previously well oiled, was introduced through the canula, to be left in the trajet of the instrument, for the purpose of rendering the canal fistulous.

"I am not sure whether it would not have been better to have postponed this perforation in the cheek until the cicatrization of the sore, which is going on rapidly, was further advanced, but I was desirous before parting with you, to take this opportunity of showing you the mode of treating salivary fistulæ, which frequently prove an obstacle to the cure of wounds in this situation. It was a proposal, I believe of Dessault's, in cases where these fistulæ prove troublesome, to compress the parotid gland on the affected side, so as to destroy its secreting power; this, however, is a practice of which I have no experience, and there are, I should suppose, few patients who could bear the necessary pressure."

In cases of salivary fistula from obstruction of the duct, the operation is frequently performed by some of our first surgeons here in the following manner. A straight needle, bearing a pretty strong ligature, armed with a knot at the end, is passed through a piece of adhesive plaster, which is arrested and hangs at the knot. The needle is introduced along the fistula as far as it will go, and then pushed through the membrane of the mouth, when it is seized and drawn out by means of a pair of forceps. The needle being now cut off, the surgeon takes hold of the ligature, and, bringing the plaster into close apposition with the external wound, fastens the other end round one of the teeth, or in some other convenient manner. A double object is thus obtained; for not only does the ligature operate as a seton, but the plaster being applied to the wound, tends to close it, which closure is promoted by lightly touching the edges every other day with the nitrate of silver. The ligature should remain for a fortnight or thereabouts.

This mode of operation was, of course, not so applicable to Dr. Ballingall's case, as to those where the fistula depends on obstruction of a salivary duct.

II. POLYPUS UTERI.—Operations on the uterus are at present quite the fashion, surgeons and physicians cutting out the womb with as much sang-froid, as if it were a mere encysted tumour. We shall shortly devote an article to the subject of these amputations, many of which seem to us hastily advised and rashly encountered. The present case is one of a common, and, therefore, an interesting affection, readily relieved without the necessity for a dangerous or bloody operation.

*Case* 3. Margaret Horn, 45 years of age, was admitted with the following symptoms. In the inside of the vagina was a large and moveable conical tumour, the apex of which might be seen from the os externum. Its consistence was soft, its colour a reddish grey, and its surface smooth, and free from ulceration. The fingers could with difficulty be passed beyond the base, by the centre of which it was connected with the surrounding parts, which, however, as well as the os tincæ, were beyond the fingers' reach. The complexion was sallow—the pulse 74—the tongue a little furred—the bowels costive—the appetite bad, and she felt great debility. The disease had commenced nearly three years before, without evident cause, and was attended with an almost habitual discharge of blood, and constant pain in the loins and side. After violent exertion, the tumour had, at times, protruded externally, though not within the last twelve months. She had borne ten children, and miscarried about the time that the tumour first appeared.

By the advice, and with the assistance, of Dr. Hamilton, the neck of the tumour was surrounded with a ligature, on the 21st of June. Slight febrile symptoms ensued, with pain in the loins and retention of urine, the latter most probably depending on the swelling of the tumour. For six successive days the ligature was tightened daily, and, on the seventh, the tumour came away, in a shrivelled and semi-gangrenous state. A weak solution of the chloride of soda was regularly thrown up the vagina, to correct the fetid and offensive discharge. The polypus, in its collapsed state, weighed eighteen ounces, and had obviously been attached by a narrow pedicle.

"The ligature has very generally been admitted to be the most eligible means of removing these polypi of the womb, and I explained to you the contrivances recommended in the works of Levret, of Desault, of Herbiniaux, of Mayer, and of Grainger, for passing ligatures around them. I pointed out the great advantages of having two canulæ, of the same dimensions, so contrived as to be separated or united at pleasure by means of additional short canulæ slipping over them. By this instrument, a ligature may be certainly carried round the base of those tumors in cases when, otherwise, it is difficult to accomplish."

Nothing can be easier, in general, than applying a ligature round the base of a polypus, by means of the double canula. It is infinitely preferable to the plan so commonly pursued in France, of drawing down the tumour to the os externum, and removing it by means of a cutting instrument. If the polypus be large, the pain and the difficulty of drawing it down are extreme, and the perineum is in danger of laceration; if small, there is no necessity for attempting it.

A case of lithotomy is detailed, which, presenting no particular features of interest, we omit. The instruments employed were a straight knife, nearly resembling a common scalpel, and a "crooked" staff, with a groove in the intermediate space between its lateral and convex faces. After the removal of the stone, Mr. Liston introduced an elastic gum tube through the wound into the bladder, a precaution to which he attributes much of his success.

III. POPLITEAL ANEURISM—*Operation.*—James Laing, ætatis 29, was admitted on the 18th of May, with an aneurismal tumour the size of an orange in the left ham. Four months previously, the swelling had commenced, and seventeen months before his admission, he had undergone the operation for aneurism in the other ham. His health was good; he was a plumber by trade, and accustomed, at times, to lift heavy weights.

On the day of his admission, the femoral artery was tied by Mr. Liston, and the pulsation in the tumour immediately arrested. On the succeeding morning, the pulsation returned in the tumour, but gradually again subsided. The ligature came away upon the 3d of June, and the wound soon healed. The patient, however, was prevented at the time from leaving the hospital, by an attack of inflammation in the site of the aneurism in the other ham. The small remains of the swelling there, at one time appeared disposed to suppuration, and were poulticed accordingly. Suppuration, however was averted; a blister was applied to promote the dispersion of the tumour, and the patient was dismissed, cured, in the month of July.

As Dr. Ballingall observes, the formation of an aneurismal tumour in one ham, soon after a cure being accomplished in the other, argues the existence of an aneurismal diathesis, and promises but poorly for his ultimate safety. There are cases, however, on record, where, under these circumstances, a permanent cure has taken place, the operation in both limbs being perfectly successful. If we do not mistake, an instance of the kind occurred, at St. George's Hospital, to Sir Everard Home, and is recorded in Mr. Hodgson's Treatise on the Arteries.

A circumstance favourable, as regards the immediate success of the operation itself, unfavourable in looking to the ultimate results, is the comparative youth of the present patient. Two aneurismal tumours at 29, argue, indeed, a disposition to the disease!

"The diminution of the tumor in the ham was more than usually rapid, and appears to me to afford a good illustration of the advantages of early operation, and of applying to the treatment of aneurism the maxim which Mr. John Bell has so energetically inculcated in reference to other tumors: 'Permit no tumor to grow uncontrolled, or to attain a dangerous magnitude.' You will not be surprized, Gentlemen, at my pressing this upon your notice, when I state that upon one occasion, within a period of twelve months, I saw three aneurisms run on to suppuration after the artery had been tied up: in two of these cases, after much suffering, the limbs were amputated, and the patients ultimately died; the third, after a tedious and extensive suppuration in the sack, was saved with great difficulty."

As we previously observed, we think the patient's age had as much, if not more, to do with the cure, as the size of the tumour itself. This, at any rate, is certain, that though, cæteris paribus, an aneurism of large size is more to be dreaded than one that is small, yet the former will often do exceedingly well after the performance of the operation.

"Many no doubt will recollect a case in illustration of this, which was in the hospital last autumn; it was that of a post-boy from Dalkeith, whose disease had been mistaken for rheumatism, and who had in consequence been treated by blistering and other measures equally inexpedient. At the time of his admission, it was one of the largest aneurisms which I had ever seen in this situation, and its fluid contents appeared to be very near the surface. Despairing of curing the disease by the common operation, and influenced perhaps by the result of the three cases to which I have just alluded, my own inclination was to have amputated the man's limb. Yielding, however, to the better judgment of my colleagues, I tied up the artery, and had the satisfaction of accomplishing a cure so as to enable the patient to resume his occupation as a postillion."

A student suggested to the Doctor, "that the common occurrence of popliteal aneurism in post-boys, might depend upon the use of the leather guard, with which they protect their leg from the pole of the carriage, and which is tightly buckled on below the knee, thus causing some interruption to the circulation of the limb." To this the Doctor *naïvely* replied, that before going farther into the speculation, he should see that the right ham only was affected, the right leg, not the left, being used in this manner. The question is really of very little consequence, the difference about as great as that "'twixt tweedledum and tweedledee!"

Dr. Ballingall concludes his report, by a general return of the surgical cases under his treatment, from the 1st of May to the end of July, in the Royal Infirmary. This, although styled by the venerable Jackson, "the common sense of common men," we shall pass altogether, numerical returns being mostly a dry and barren method of communicating information. Dr. Ballingall, we suspect, has a dash of the "man of sentiment" about him, for he generally closes his report with a moral or professional precept. We wish the following were practised as earnestly as it is taught.

"I should wish above all things to see you communicating the final results of cases which may have been the subjects of surgical operation, in order that we may be encouraged to persist in the practice of those operations which afford permanent relief and general satisfaction; and that we may not be hurried by a premature or imperfect disclosure of facts to perform operations which necessarily subject the patient to present suffering, subsequent delusion, and ultimate disappointment."

We tender Dr. B. our best thanks for his report, which is ably and honestly drawn up.

